# A case report of gastric amyloidosis due to multiple myeloma mimicking gastric cancer

**DOI:** 10.1186/s12876-020-01359-z

**Published:** 2020-07-11

**Authors:** Huini Xiao, Duxin Qing, Chenjie Li, Hejun Zhou

**Affiliations:** Department of Gastroenterology, Second Xiangya Hospital of Central South University, Changsha, 410000 China

**Keywords:** Gastrointestinal amyloidosis, Multiple myeloma, Gastric cancer, Congo red staining, Bone marrow biopsy

## Abstract

**Background:**

Gastrointestinal (GI) amyloidosis is a rare complication of multiple myeloma (MM). Due to its nonspecific clinical presentation and endoscopic appearance, an early and accurate diagnosis of GI amyloidosis is difficult. Here, we report a case of GI amyloidosis due to MM, which initially presented as GI manifestations mimicking gastric cancer.

**Case presentation:**

A 68-year-old woman presented to the hospital with a 6-month history of anemia, coupled with a recent onset of poor appetite and vomiting for 10 days. Esophagogastroduodenoscopy revealed a gastric antrum mucosal bulge that appeared on visual inspection to be a tumor. As a result, gastric cancer was suspected. However, gastric antrum biopsies demonstrated mild chronic superficial gastritis and esophageal biopsies demonstrated moderate-to-severe atypical hyperplasia of the squamous epithelium. A second endoscopy revealed massive gastric retention and a gastric antrum mucosal bulge with surface erosion. Ultimately, an upper GI tract biopsy demonstrating positive Congo red staining and a bone marrow biopsy indicating plasmacytosis confirmed the diagnosis of gastric amyloidosis due to MM.

**Conclusion:**

This case demonstrates that MM should be considered in patients with nonspecific GI manifestations, and in such cases, a biopsy with Congo red staining should be considered to confirm GI amyloidosis. Early detection of GI amyloidosis will ultimately improve outcomes for these rare patients.

## Background

Multiple myeloma (MM) is the most common type of multifocal plasma cell proliferation in the bone marrow and is associated with the overproduction of immunoglobulins. Renal failure, anemia, skeletal lesions, and recurrent infections are the most common clinical manifestations of the disease [1]. Gastrointestinal (GI) amyloidosis is a rare and complex complication of MM. Only a small number of cases describing amyloidosis-induced gastrointestinal complications as the presenting symptom of MM have been reported [2–11]. Furthermore, previous studies have not described the many similarities between gastric amyloidosis and gastric cancer, including the clinical presentation and both the endoscopic and microscopic appearances. Therefore, alimentary symptoms may be easily ignored, which can increase the rates of misdiagnosis and missed diagnosis. In this study, we report an unusual case of gastric amyloidosis due to MM mimicking gastric cancer. Our hope in doing so is to increase the index of suspicion of both the physician and pathologist for the early detection of GI amyloidosis.

## Case presentation

**Fig. 1:**
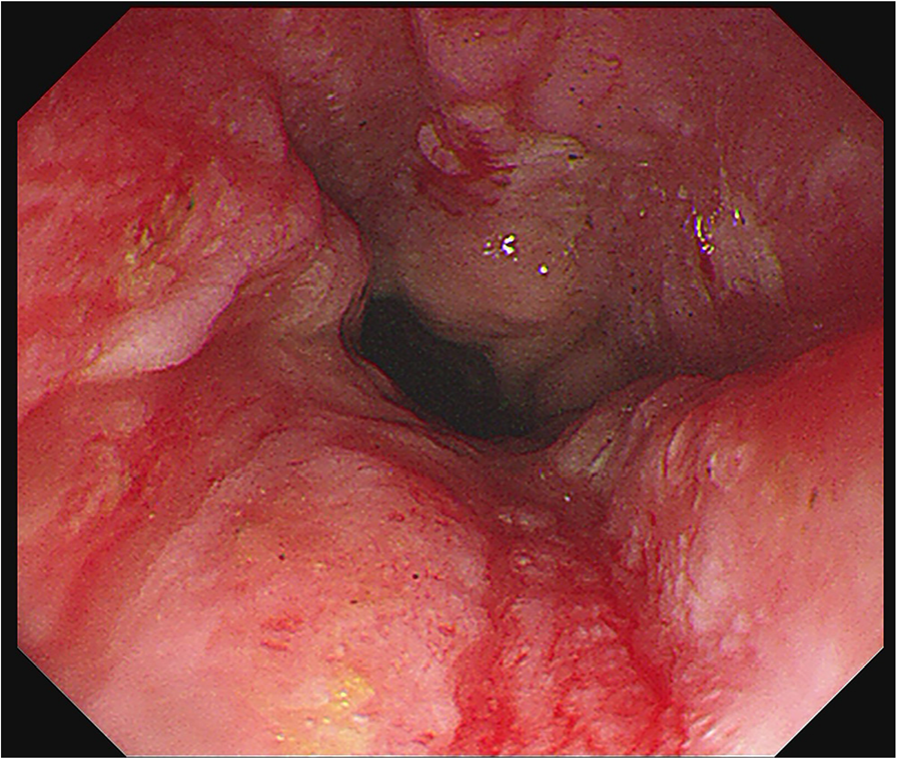
Esophageal endoscopic image obtained during the patient’s first EGD demonstrating congestion, swelling, roughness, and erosion of the middle and lower esophageal mucosa

**Fig. 2:**
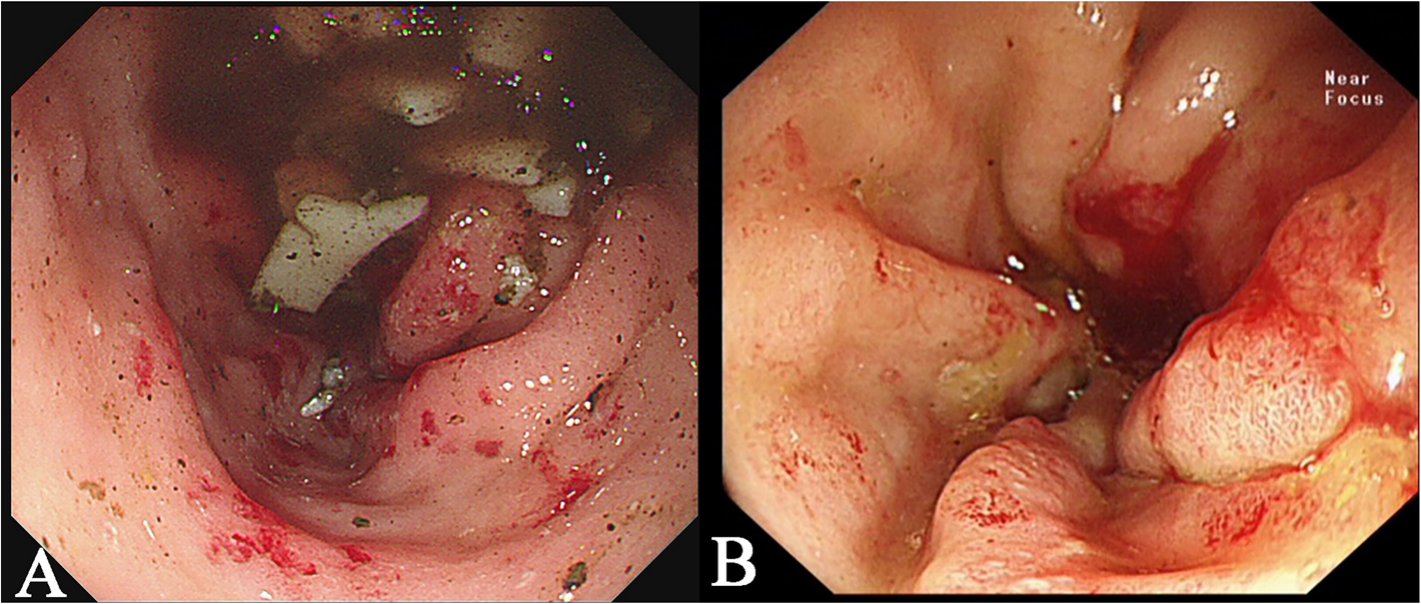
Gastrointestinal endoscopic images revealing: **a**, gastric antrum mucosal nodular uplift with erosion and pyloric stenosis (image obtained during the patient’s first EGD); **b**, gastric antrum mucosal bulge with erosion and incomplete obstruction (image obtained during the patient’s second EGD)

A 68-year-old woman presented to the hospital with a 6-month history of anemia coupled with a recent onset of poor appetite and vomiting for 10 days. She also had a history of lumbar disc herniation. Initial biochemical investigations revealed a hemoglobin level of 8.0 g/dL, serum creatinine level of 2.21 mg/dL, and corrected calcium level of 2.74 mmol/L. Liver function was normal, but albumin level was 29.5 g/L (normal range: 40–55 g/L) and globulin level was 45 g/L (normal range: 20–40 g/L). Moreover, fecal occult blood testing was positive. Lung computed tomography demonstrated thickening of the esophageal wall and multiple enlarged mediastinal lymph nodes. Abdominal sufficiency computed tomography demonstrated thickening of the gastric wall and gastric retention. Esophagogastroduodenoscopy (EGD) revealed congestion, swelling, roughness, and erosion of the middle and lower esophageal mucosa (Fig. 1), mucosal nodular uplift with erosion in the gastric antrum, tube wall stiffness, and pyloric stenosis, suspecting gastric antrum cancer combined with incomplete obstruction (Fig. 2a). Endoscopic ultrasonography was not appropriate for this patient due to her poor overall condition as well as the large amount of retention in her stomach, which would adversely affect the results of the examination.

**Fig. 3:**
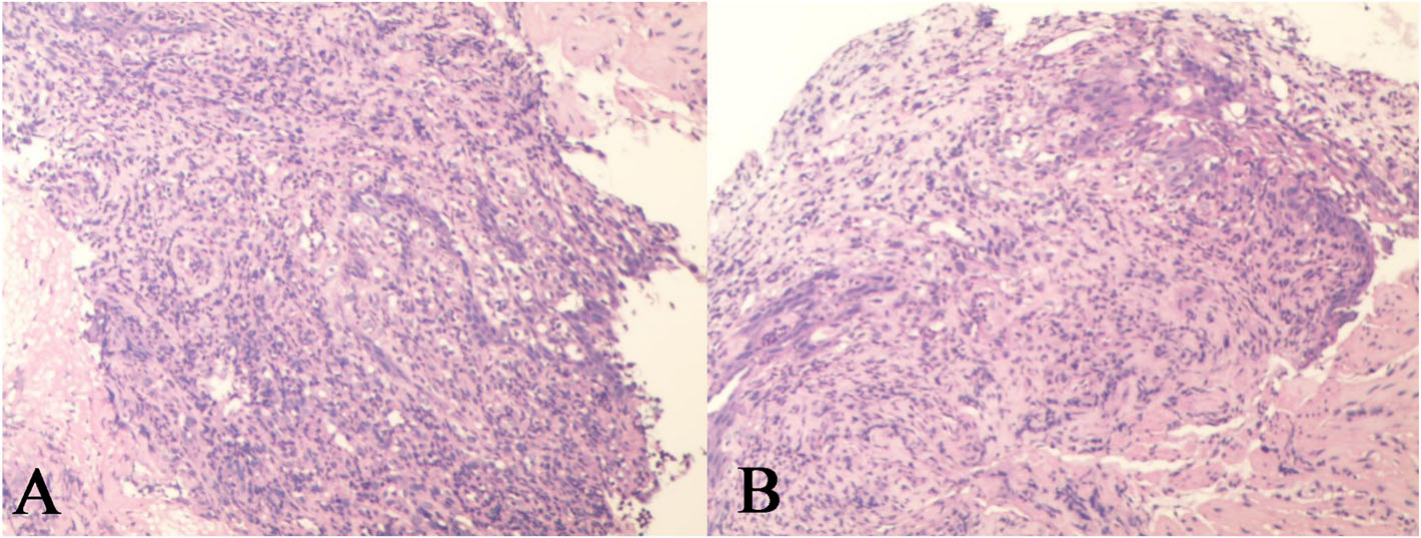
Histopathological findings of esophageal biopsies obtained during the patient’s first EGD revealing moderate-to-severe atypical hyperplasia of the squamous epithelium

The patient’s clinical presentation and results of her evaluations first led us to suspect a diagnosis of gastric cancer. However, biopsies taken from the gastric antrum demonstrated mild chronic superficial gastritis, and biopsies taken from the esophagus demonstrated moderate-to-severe atypical hyperplasia of the squamous epithelium (Fig. 3).

**Fig. 4:**
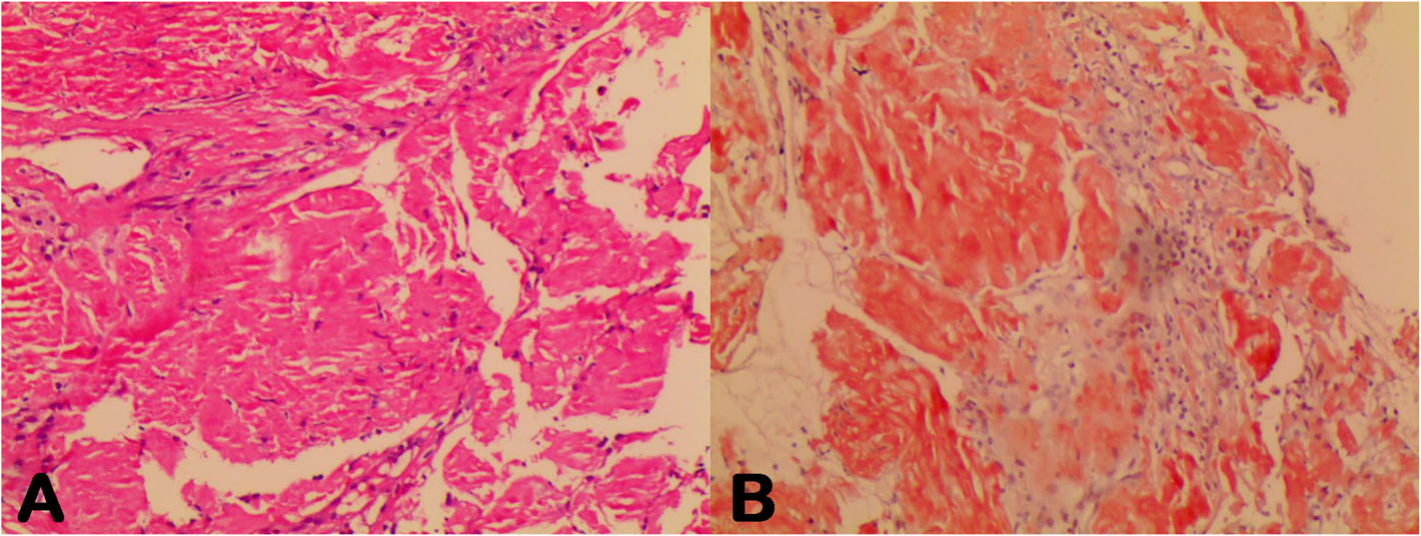
Histopathological findings of stomach biopsies obtained during the patient’s second EGD revealing: **a**, amyloidosis in the gastric antrum, (100×); **b**, positive Congo red staining in the gastric antrum, (100×)

**Fig. 5:**
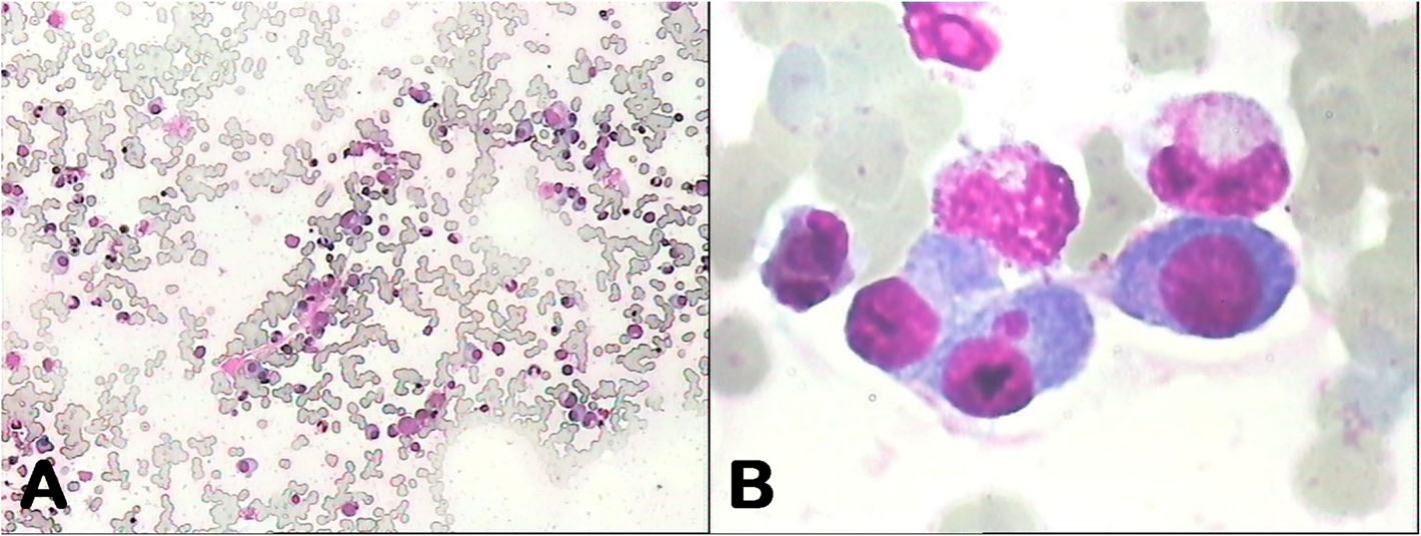
Bone marrow analysis: a, cytological examination; b, abnormal plasma cells characterized by their large nucleus and identified as myeloma cells

To clarify the diagnosis, the patient underwent a second EGD, which confirmed a large amount of food retained in the stomach. In addition, the mucosa of the gastric fundus, stomach body, gastric angular, and gastric antrum were all hyperemic and swollen, and a gastric antrum mucosal bulge with surface erosion was noted (Fig. 2b). Histopathological examination indicated an extensive deposition of hyaline amorphous eosinophilic extracellular material and mucosal biopsies from both the stomach and esophagus stained positive on Congo red staining (Fig. 4). No evidence of gastric neoplasia was detected. A colonoscopy was not completed because of the patient’s poor general condition. Immunoglobulin G (IgG) level was 44.8 g/L (normal range: 6–16 g/L). Immunofixation and immunophenotyping of IgG and λ chain yielded positive results. X-ray revealed degeneration in both the sacroiliac and hip joints and a compression fracture in the first vertebra. Moreover, a bone marrow biopsy revealed that atypical clonal plasma cells accounted for 34% of the plasma cells (Fig. 5). Based on these examination results, a definite diagnosis of gastric amyloidosis due to MM was confirmed. Regrettably, the patient died due to multiple organ failure before chemotherapy could be initiated. Earlier diagnosis of this patient’s underlying disease may have allowed chemotherapy to be initiated.

## Discussion and conclusions

MM is a malignant disease characterized by clonal plasma cell proliferation in the bone marrow and is most common in the elderly [12]. Amyloidosis is a group of diseases that involve the deposition of amyloidogenic proteins in various organs and can ultimately lead to multiple organ failure [13]. More than 40 extracellular proteins have been identified to form amyloid in humans so far. Light-chain (AL) amyloidosis is classified as primary amyloidosis or amyloidosis secondary to myeloma [14], and it is the most common form of systemic amyloidosis. Approximately 10–15% of patients with MM develop overt AL amyloidosis. The incidence of AL amyloidosis significantly increases with age. It is often associated with proteinuria, cardiac amyloidosis, and peripheral neuropathy [15]. Cowan et al. reported results from a 13-year, single-center, referral experience, which found that 76 patients (3.2%) had biopsy-proven amyloid with gastrointestinal involvement among 2334 patients with all types of amyloidosis [16]. Among patients with systemic amyloidosis, gastrointestinal tract involvement is very common [17]. However, only a small number of cases describing amyloidosis-induced gastrointestinal complications as the presenting symptom of MM have been reported [2–11]. In a review from the Mayo Clinic, symptomatic gastrointestinal amyloidosis had a 1% incidence in primary systemic amyloidosis [18]. Therefore, gastrointestinal symptoms may be clinically ignored. In addition, most clinical presentations involve nonspecific GI symptoms such as abdominal pain, hemorrhage, intestinal perforation, motility disorders, and malabsorption. The most common endoscopic presentations of AL type amyloidosis within the luminal gastrointestinal tract are mucosal erosions, ulcerations, and submucosal hematomas [19], and fine granular and polypoid protrusions [20]. Amyloid deposits may also cause recurrent peptic ulcers; thus, gastric amyloidosis may also present as gastric dysplasia.

It remains a challenge to draw an accurate diagnosis of MM. In our case, anemia, alimentary symptoms, and melena were the chief complaints. EGD revealed gastric retention and a gastric antrum mucosal bulge with surface erosion. Because of these misleading manifestations, we misinterpreted the signs of a systemic disease as including skeletal Rx-lesions, renal failure, and higher circulating immunoglobulin levels. The first pathological examination without special staining initially indicated chronic superficial gastritis (mild). However, follow-up pathological examination led to a definite diagnosis of gastric amyloidosis through positive Congo red staining and bone marrow biopsy.

For patients with digestive system symptoms and nonspecific endoscopic features, it is important to consider the diagnosis of amyloidosis and a biopsy with Congo red staining should be performed for the precise diagnosis for GI amyloidosis. After amyloidosis is confirmed, immunoglobulin free light chain κ and λ testing, bone marrow biopsy, serum and urine immunofixation, echocardiography, 24-h urine total protein test, and quantitative immunoglobulin measurement should be performed [21] to assess the underlying disorder. The treatment and prognosis of amyloidosis depend on whether patients have systemic or localized disease. The severity of cardiac dysfunction at the time of diagnosis is a determinant of the survival of patients with systemic AL amyloidosis [21, 22].

Drug therapy for AL amyloidosis includes conventional chemotherapy such as melphalan or prednisolone and some novel drugs that include proteasome inhibitors and immunomodulatory drugs [23]. For most AL patients, the pathological clone of plasma cells is relatively benign and various drugs can be effective. Nevertheless, they are often poorly tolerated because of damaged organ function. Thus, as reported in this case, treatments for the elderly are delayed because of their poor general condition that does not allow chemotherapy to be performed. One retrospective study found that the time from symptom onset to diagnosis was 7–24 months [24]. Part of the reason for this delay is the overall rarity of the disease. Early accurate diagnosis is possible if doctors can associate nonspecific clinical symptoms with multiple systemic data and establish close communication with pathologists.

In conclusion, this case highlights the importance of a clinical suspicion of gastric amyloidosis in patients with nonspecific GI symptoms such as vomiting or melaena. In addition, endoscopic biopsy with Congo red staining should be performed for differentiating between amyloidosis and cancer because of the similar endoscopic features of the two condition. Early diagnosis and treatment are essential for patients with gastric amyloidosis because of its severity and poor prognosis.

## Data Availability

Not applicable.

## Abbreviations

GI: Gastrointestinal
MM: Multiple myeloma
EGD: Esophagogastroduodenoscopy
AL amyloidosis: Light-chain amyloidosis
